# Supplementary material for: Complexome profiling of the Chlamydomonas psb28 mutant reveals TEF5 as an early PSII assembly factor
Source: Plant Cell. 2025 Mar 17;37(6):koaf055. doi: 10.1093/plcell/koaf055 (PMC12164590; doi:10.1093/plcell/koaf055)
Supplement: koaf055_Supplementary_Data [file koaf055_supplementary_data.zip › psb28_interactive.zip!/plots/intensity/qualityPlots/peptides/CIS1_Cre12.g514750.t1.2.html]

### Cre12.g514750.t1.2

Trivial name: CIS1  
SeqCoverage [median]: 33.00  
Mass: 51.29 kDa  
  
 

| Sequence | MissedCleavage | Mass [Da] | wtIntensity | muIntensity | Score |
| --- | --- | --- | --- | --- | --- |
| ALGLPIERPK | 1 | 1092.67 | 8521.35 | 26204.46 | 90.11 |
| ELIPAQQER | 0 | 1082.57 | 15893.87 | 21233.36 | 139.88 |
| FQSAYAR | 0 | 841.41 | 9786.84 | 15253.14 | 153.15 |
| GLSIPELQEK | 0 | 1112.61 | 2491.60 | 12573.26 | 88.37 |
| KYVEDTLASGK | 1 | 1209.62 | 5655.04 | 10232.81 | 112.13 |
| LGPSPGRDAVR | 1 | 1123.61 | 0.00 | 756.46 | 41.40 |
| LPQLAATIYR | 0 | 1144.66 | 2007.33 | 8921.90 | 88.95 |
| LVADLYEVVPEVLGK | 0 | 1642.92 | 1958.31 | 0.00 | 184.03 |
| SLGEVTVDMAIGGMR | 0 | 1534.75 | 6160.16 | 19325.13 | 190.53 |
| SLTMAALEQR | 0 | 1118.58 | 13303.57 | 17735.45 | 210.60 |
| SNVPLHLR | 0 | 934.53 | 0.00 | 1374.71 | 79.12 |
| SNVPLHLRK | 1 | 1062.63 | 2063.92 | 4261.66 | 66.27 |
| VLDALPTEAHPMTQLSMGIMALQSGSR | 0 | 2853.42 | 0.00 | 3932.53 | 61.37 |
| VVPGYGHAVLR | 0 | 1166.66 | 7202.16 | 16229.74 | 91.58 |
| YVEDTLASGK | 0 | 1081.53 | 6976.78 | 7200.74 | 106.88 |
|  |  |  | 82020.9 | 165235.3 |  |
